# Isolation and identification of a novel protein elicitor from a *Bacillus subtilis* strain BU412

**DOI:** 10.1186/s13568-019-0822-5

**Published:** 2019-07-27

**Authors:** Yongrui Shen, Jianwei Li, Junliang Xiang, Jiaqi Wang, Kuide Yin, Quan Liu

**Affiliations:** College of Life Science and Technology, Heilongjiang Bayi Agricultural University, Daqing, 163319 China

**Keywords:** Protein elicitor, *Bacillus subtilis*, Hypersensitive response, Reactive oxygen species, Induced systemic resistance

## Abstract

Here, we report a novel protein elicitor from *Bacillus subtilis* BU412 which could cause hypersensitive response (HR) and systemic acquired resistance (SAR) in tobacco. The purification was executed by ion-exchange and size exclusion chromatography. The target band on SDS-PAGE was analyzed by mass spectrometry, and the peptide mass fingerprinting matched an uncharacterized protein (WP_017418614.1), which was then named AMEP412. AMEP412 could cause a clearly defined HR necrosis in tobacco leaves, which was less affected by thermal treatment. The sub-cellular localization assay revealed that AMEP412 localized on the cell surface. This protein could also trigger early defense events such as the generation of reactive oxygen species (H_2_O_2_ and O_2_^−^) and the induction of defense enzymes, including superoxide dismutase (SOD), peroxidase (POD), polyphenol oxidase (PPO) and phenylalanine ammonia-lyase (PAL). Moreover, AMEP412 could stimulate plant systemic resistance against *Pseudomonas syringae* pv. *tomato* DC3000.

**Electronic supplementary material:**

The online version of this article (10.1186/s13568-019-0822-5) contains supplementary material, which is available to authorized users.

## Introduction

During the long term interaction with pathogens, plants evolved different regulatory mechanisms to escape the attacks from pathogens (Díez-Navajas et al. 2008; Pieterse et al. 2009; Dodds and Rathjen 2010). Recognition of the pathogens or other foreign molecules is critical for the initiation of defence responses (Bruce and Pickett 2007). Elicitors, produced and released by microbes, are thought to have significant roles in signal exchange between plants and pathogens (Mishra et al. 2012).

Elicitors can induce plant defence responses, such as cell wall strengthening, reactive oxygen species (ROS), ethylene biosynthesis, expression of pathogenesis-related (PR) proteins, and induction of hypersensitive response (HR) (Wang et al. 2004; Miyata et al. 2006; Wang et al. 2012). These responses are first expressed in the infected area, which is called induced system resistance (ISR), and then extend to the non-infected area and create a systemic acquired resistance (SAR) (Yano et al. 1998; Durrant and Dong 2004; Garcia-Brugger et al. 2006).

Many protein elicitors have been isolated from a variety of pathogens, including Flagellin and Harpin from bacteria (Che et al. 2000; Wei et al. 1992), xylanase from fungi (Hanania and Avni 1997), invertase from yeast (Basse et al. 1993), and Elicitins from oomycetes (Ricci et al. 1989). However, several protein elicitors from biocontrol strains also have been reported to induce disease resistance, like Fengycins and Surfactins from *Bacillus subtilis* (Ongena et al. 2007), PeBA1 from *Bacillus amyloliquefaciens* (Wang et al. 2016) and BAR11 from *Saccharothrix yanglingensis* (Zhang et al. 2018).

In this paper, we reported the purification and characterization of a novel protein elicitor from a biocontrol strain *Bacillus subtilis* BU412. We performed a purification process that consisted of ion-exchange and size exclusion chromatography to gain the new protein elicitor, and identified it by mass spectrometry. Plant defence response caused by the new protein elicitor was determined, like HR, ROS burst, induction of defense enzymes, and SAR against infection by *Pseudomonas syringae* pv. *tomato* DC3000.

## Materials and methods

### Plants, strains, and growth conditions

*Nicotiana tabacum* was grown at 24–26 °C, with a 12-h light/dark in a phytotron. *Bacillus subtilis* BU412 was isolated from potato field by our laboratory and deposited in China Center for Type Culture Collection (CCTCC M2016142). YME medium (Schaad et al. 2001) was used to culture the strain. *Pseudomonas syringae* pv. *tomato* (Pst) DC3000 (ATCC BAA-871) was cultured in low salt LB (half dosage of NaCl) medium containing 50 mg L^−1^ rifampicin at 28 °C overnight as previously described (Katagiri et al. 2002). Culture cells were harvested and the final concentration of cell suspensions was adjusted to 5 × 10^5^ cfu mL^−1^ using 20 mM Tris–HCl (pH 7.5).

### Protein purification

A single colony of *Bacillus subtilis* BU412 was cultured in YME liquid medium at 32 °C and 160 rpm for 12 h as seed liquid. 3 mL seed liquid was inoculated into 300 mL YME liquid medium and cultured at 32 °C, 160 rpm for 22 h. The supernatant was collected by centrifugation at 4 °C, 16,000×*g* for 30 min.

The culture supernatant was filtered through 0.22 μm membrane and applied to a Source 15Q 4.6/100 PE column, on an AKTA Purifier system (Amersham Biosciences) pre-equilibrated with 20 mM Tris–HCl (pH 7.5). The column was washed with a linear gradient of 0.5 M NaCl from 0 to 100% concentration in 20 mM Tris–HCl (pH 7.5) at a flow rate of 1 mL min^−1^. Individual peak fractions were concentrated to 1 mg mL^−1^ by Amicon ultra centrifugal filters (Millipore) and tested for HR activity on tobacco leaves. Protein samples with HR activity were applied to a Superdex 75 10/300 GL column. The column was eluted with 20 mM Tris–HCl (pH 7.5) at a flow rate of 0.8 mL min^−1^. Fractions were collected and tested for HR activity, and then determined by SDS-PAGE. All purification steps were performed at room temperature, and the column effluent was monitored by absorbance at 280 nm.

### Mass spectrum analysis

The exclusive protein band in SDS-PAGE gel was removed, gel-digested and analyzed with 4700 MALDI-TOF/TOF mass spectrometer (AB SCIEX). Strong mother ions were selected to get second mass spectrum (MS/MS). The peptide mass fingerprinting (PMF) of MS and MS/MS was searched with Mascot (Matrix Sciences) search engine to identify the protein.

### Characterization of the elicitor

Protein elicitor samples used in the following assays were purified from the supernatant of BU412 culture through ion-exchange and size exclusion chromatography following the methods mentioned above. All protein concentrations were measured using NanoDrop One UV Spectrophotometer (Thermo Scientific).

#### HR and Trypan blue staining

In order to check the effect of the new protein elicitor for the HR-inducing activity in tobacco, 1 mg mL^−1^ protein elicitor was infiltrated into the leaves using a syringe without needle to cover areas of 1 cm^2^. The HR symptom necrosis was examined in the injected areas after 24 h. Tobacco leaves with HR were stained by Trypan blue and then observed under a microscope, according to the previously described method (Koch and Slusarenko 1990).

#### The minimum concentration for HR induction

In order to check the minimum concentration of the new protein elicitor for the induction of HR in tobacco, different concentrations of protein elicitor (2.4, 2.0, 1.6, 1.2, 0.8 and 0.4 mg mL^−1^) in a 100-μL volume were infiltrated into tobacco leaves using a 1 mL needleless syringe, with 20 mM Tris–HCl (pH 7.5) as control. HR symptoms were examined after 24 h.

#### The thermo stability test of the protein elicitor

To test the thermo stability, protein elicitor was treated at different temperatures (25, 40, 60, 80, and 100 °C) for 5 min and then infiltrated into tobacco leaves after cooling to room temperature. The HR responses for infiltrated tobacco leaves were observed after 24 h.

### Sub-cellular localization

Two milligrams of the protein elicitor was reacted with 0.1 mg of FITC in 2 mL of carbonate buffer (0.05 M, pH 9.0) for 12 h at 4 °C. The FITC and protein elicitor mixture was applied to a Superdex 75 10/300 GL column equilibrated and eluted with 20 mM Tris–HCl (pH 7.5). Thus, FITC-elicitor was separated from free FITC molecules. Subsequently, FITC-elicitor was infiltrated into the upper leaves of 6-week-old *N. tabacum* plants using a 1 mL-syringe without needle. The leaves were shredded at 4 h post injection, and a laser confocal microscope (Leica SP8) with an excitation wavelength of 495 nm was then used to observe the localization.

### ROS accumulation

One of the early events during the HR is the generation of reactive oxygen as an active process to signal downstream cellular processes (Torres et al. 2006). Accumulation of hydrogen peroxide was detected by a peroxidase-dependent in situ histochemical staining procedure using 3,3-diaminobenzidine (DAB) (Thordal-Christensen et al. 1997) and superoxide ion using a superoxide-dependent reduction of nitro blue tetrazolium (NBT) (Doke 1983). Leaves of *N. tabacum* plants were sprayed with 50 μg mL^−1^ AMEP412, and buffers were used as control. At different post treatment hours (0, 4, 12, and 24 h), leaves were cut and then vacuum-infiltrated with 1 mg mL^−1^ DAB (pH 3.8) or 1 mg mL^−1^ NBT for 2 h. The treated leaves were incubated for more than 24 h in 70% ethanol and 5% glycerol to eliminate chlorophyll, observed for DAB and NBT deposits, and photographed.

### Induction of defense enzymes

Leaves of *N. tabacum* plants were sprayed with 50 μg mL^−1^ AMEP412, and buffers were used as control. Leaves were harvested at different times (0, 4, 8, 12, 24, 48, and 72 h) after treatment and immediately frozen in liquid nitrogen. Then, samples of each treatment were homogenized in extraction buffer (50 mM phosphate buffer, pH 7.8) using mortar and pestle. The lysate was then centrifuged at 16,000×*g* for 20 min at 4 °C. The supernatant was collected for use as crude enzyme extracts. The activities of SOD, POD, PPO and PAL were assayed according to the previously described method (Hano et al. 2008).

### Induced disease resistance in tobacco

Six-week-old *N. tabacum* plants were used for the following assay. Two leaves of tobacco plant were treated with protein elicitor (50 μg mL^−1^) by spraying, using buffer as control. At 24 h post treatment, 50 μL Pst DC3000 cell suspension (5 × 10^5^ cfu mL^−1^) was infiltrated into the untreated systemic leaves using a 1 mL-syringe without needle. Inoculated plants were maintained in growth chamber at 22 °C with high humidity and a 16-h day/8-h night cycle. Symptoms were observed 4 days post infection with Pst DC3000.

## Results

### Purification and identification of the new protein elicitor

**Fig. 1:**
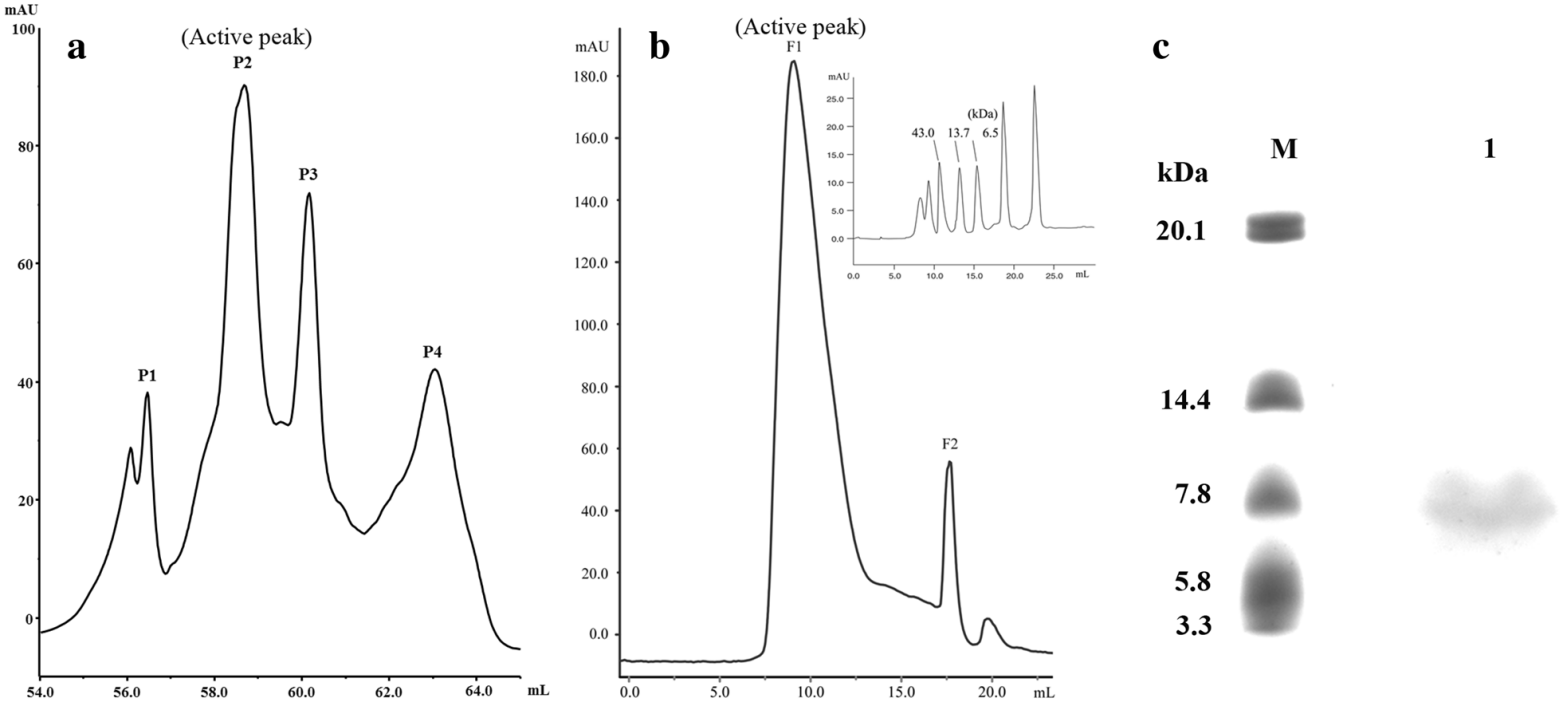
The purification of the new elicitor protein. **a** The anion exchange chromatography map. P1-P4 were peaks eluted with a linear gradient of NaCl. **b** The superdex chromatography map. F1 and F2 were peaks eluted with 20 mM Tris–HCl. Inset, typical chromatogram from a function test of Superdex 75 10/300 GL (Amersham Biosciences). **c** The SDS-PAGE detection of the target protein. M: low molecular weight standards, 1: the purified protein sample of the peak F2, B1: the target protein band

The supernatant was prepared through centrifugation and filtration, which was then applied for anion exchange purification. The anion exchange chromatography obtained four main peaks after a linear gradient elution (Fig. 1a), and the peak P2 showed HR activity. Then P2 was concentrated and further purified by Superdex column, and two main peaks were collected (Fig. 1b). The peak F1 had HR activity and showed a main megascopic band (B1) around 7 kDa on SDS-PAGE (Fig. 1c), which was thought to be the target protein.

Band B1 was cut off and analyzed by Maldi-TOF mass spectrometer and the mass spectrum was shown in Additional file 1: Figure S1. Mascot search results indicated that peptide mass fingerprinting (PMF) of the sequenced protein had the best similarity to an uncharacterized protein (WP_017418614.1). The amino acid sequence was shown in Additional file 1: Figure S2 with bold and italic letters indicating the matched amino acids with the sequenced protein. There were five amino acid fragments matched that included 37 amino acid residues and sequence coverage was 49%. The above results allowed us to conclude that the protein identified in this study was most likely an uncharacterized protein, which was named AMEP412.

According to the analysis result of ProtParam (Wilkins et al. 1999), AMEP412 contained 76 amino acid residues with a relative molecular mass (MW) of 8.36 kDa. In addition, the protein also consisted of 15.8% lysine, 13.2% alanine and 10.5% leucine. The protein had 2 negatively charged residues and 12 positively charged residues, and the isoelectric point (pI) reached 10.05. The instability index was computed to be 1.35, which classified the protein as stable. A secondary structure prediction server (Drozdetskiy et al. 2015) analysis indicated that AMEP412 had five á-helices with no â-sheet and random coil (Additional file 1: Figure S2). The predicted results for the transmembrane domain (Tusnády and Simon 2001) showed that there was a transmembrane domain at position 17–36, which implied its binding ability with the membrane.

### Characterization of AMEP412

**Fig. 2:**
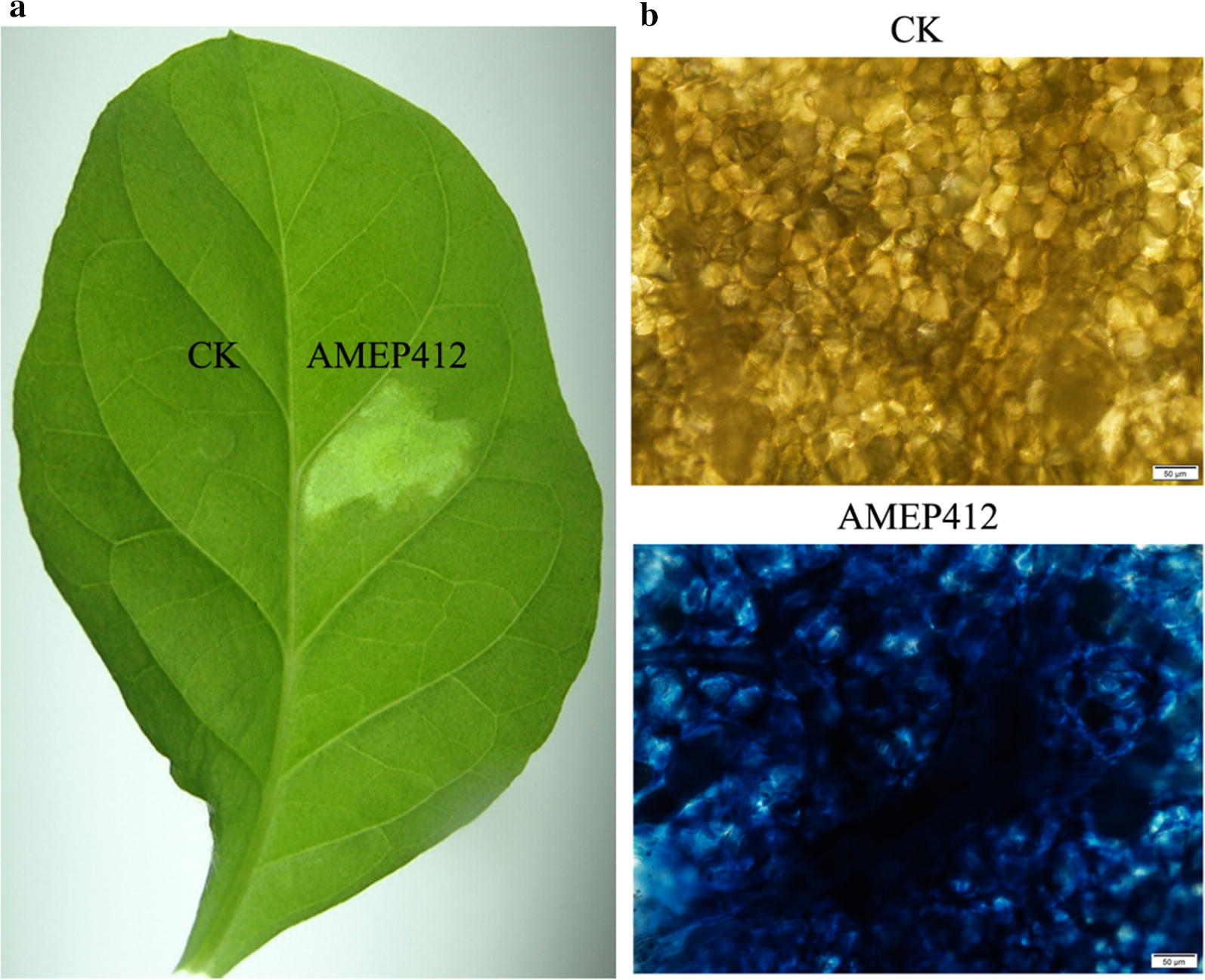
AMEP412 induced HR in tobacco leaves. **a** HR lesion caused by AMEP412 in tobacco leaves. **b** Trypan blue staining of the HR areas infiltrated by AMEP412. Buffer treatment areas could not be stained by dye. AMEP412 induced cell death in infiltrated areas was stained blue. Scale bar = 50 μm

To confirm the HR activity of the new protein, 1 mg mL^−1^ AMEP412 was infiltrated into tobacco leaves, and clearly defined HR necrotic areas were found at the infiltration sites (Fig. 2a). HR is also a kind of cell death, which can be monitored by Trypan blue staining on the leaves. In our test, dead cells located at the site of HR were stained blue (Fig. 2b).

**Fig. 3:**
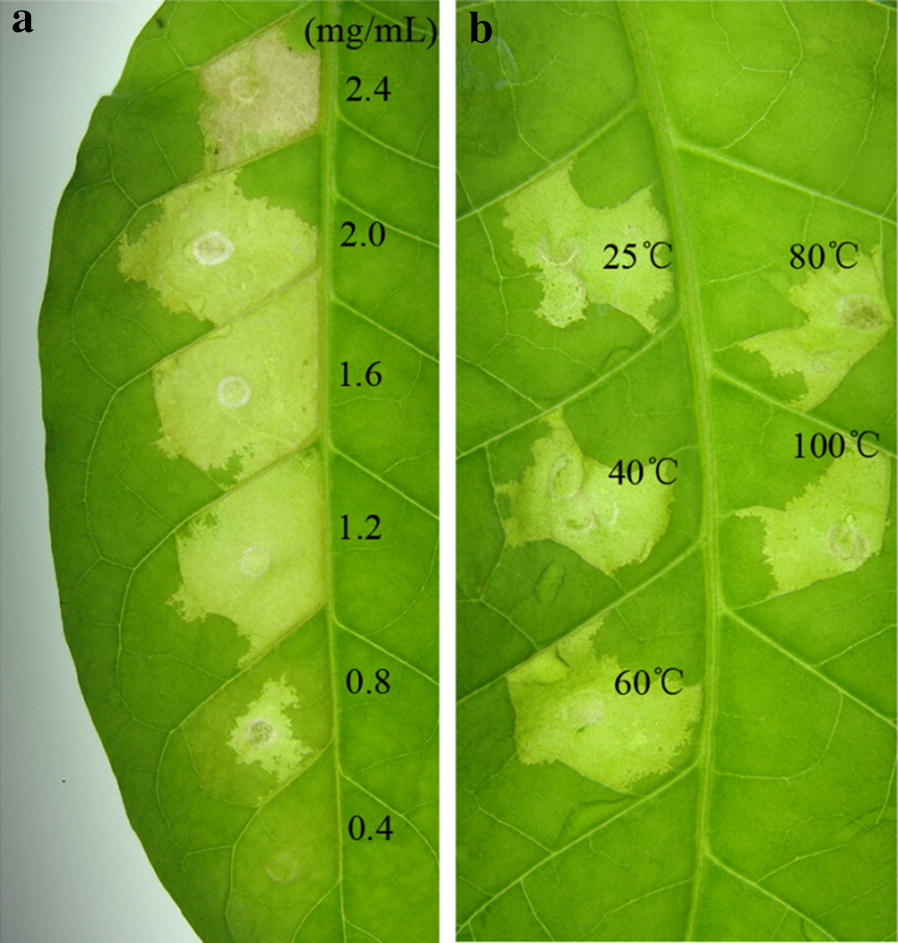
The characterization of AMEP412 for its HR activity. **a** The minimum concentration of AMEP412 for its HR activity. Serial dilutions of AMEP412 (2.4, 2.0, 1.6, 1.2, 0.8 and 0.4 mg mL^−1^) were infiltrated into tobacco leaves, and HR was observed after 24 h. **b** The thermal stability of AMEP412 for its HR activity. AMEP412 was treated at different temperatures (25, 40, 60, 80 and 100 °C) for 5 min, infiltrated into leaves and photographed at 24 h

In order to check the minimum concentration needed for HR, serial dilutions of AMEP412 were infiltrated into tobacco leaves and the results showed that the minimum concentration was 0.8 mg mL^−1^ (Fig. 3a).

The thermo stability test showed that AMEP412 could induce obvious HR symptoms after treated at 25, 40, 60, 80 and 100 °C for 5 min, which suggested that AMEP412 had good thermal stability (Fig. 3b).

### Localization assay

**Fig. 4:**
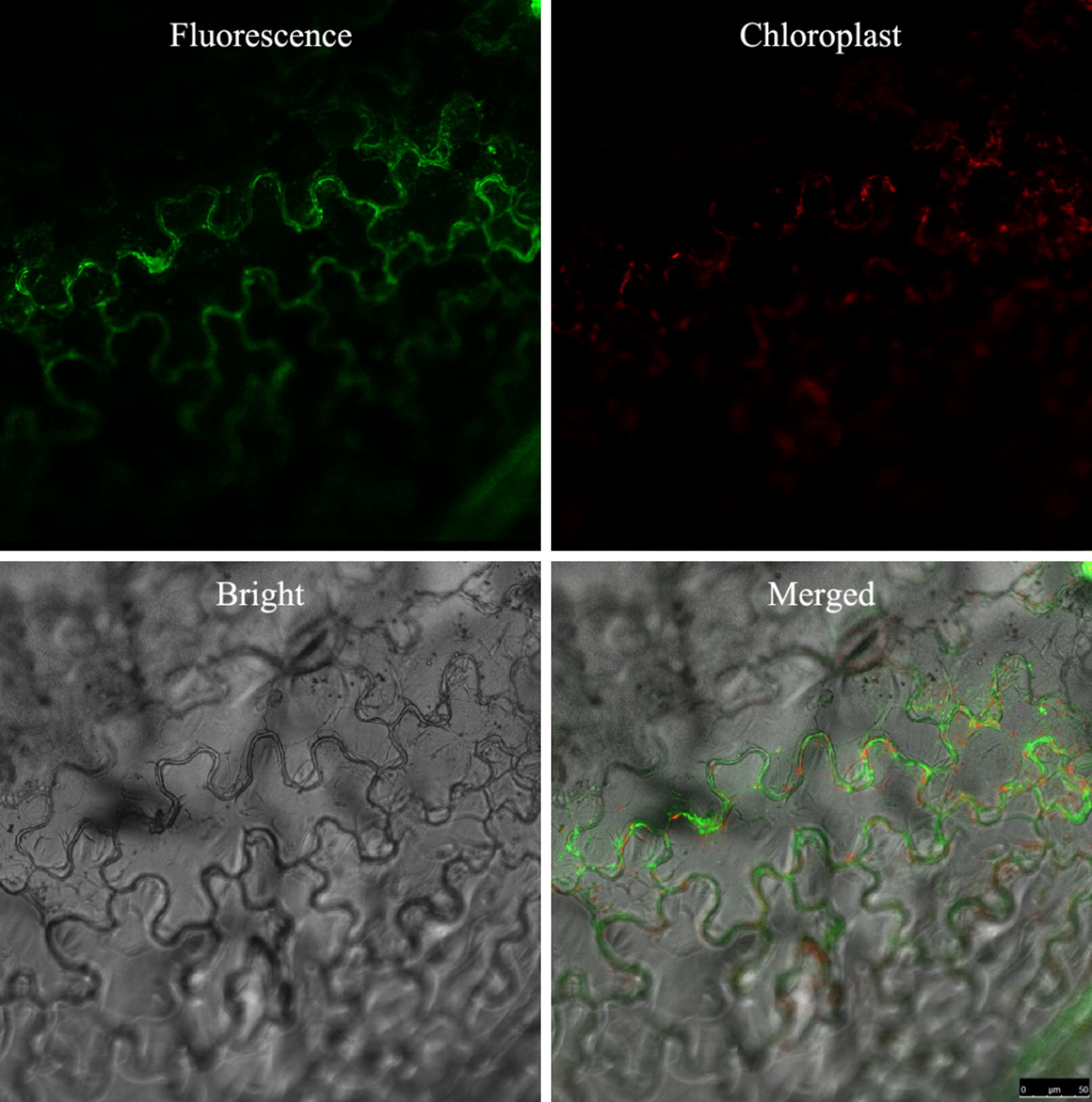
Sub-cellular localization of AMEP412 in tobacco cells

The cellular localization of AMEP412 was determined by generating a fusion with FITC. FITC-AMEP412 was infiltrated into tobacco leaves, and the fluorescent signals were observed using laser confocal microscope after 4 h incubation. As shown in Fig. 4, the fluorescence was observed to distribute almost uniformly along the cell wall and the cell peripheral surface. This result suggested that AMEP412 localized in the cell surface, which provided clues for the mechanism research.

### Induction of ROS production

**Fig. 5:**
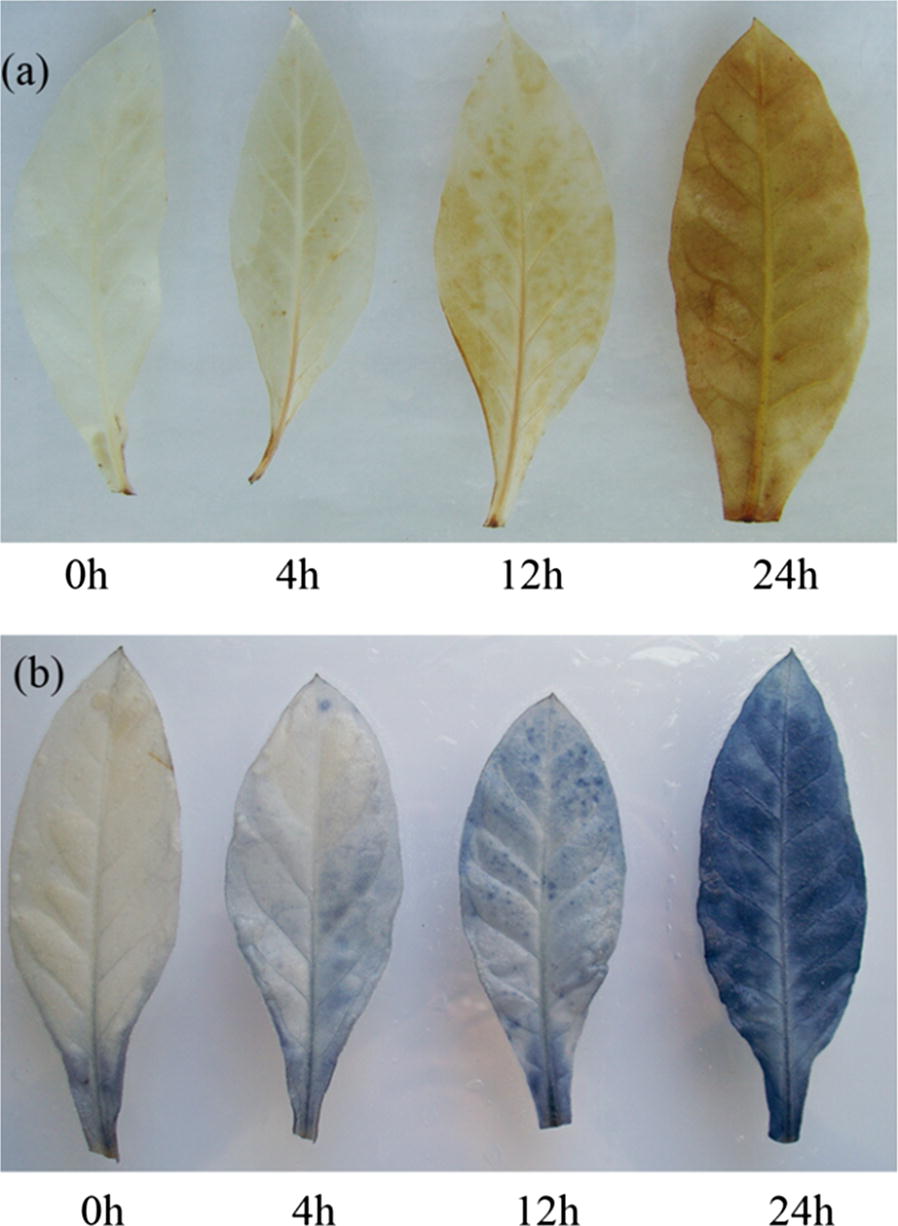
Induction of ROS in tobacco leaves by AMEP412. **a** DAB showed the production of H_2_O_2_. The leaves were stained at different post treatment hours. H_2_O_2_ accumulation appeared in AMEP412 treated leaves. **b** NBT revealed the production of O_2_^−^, and staining was performed on leaves at the same treatment time. O_2_^−^ accumulation appeared in AMEP412 treated leaves

To further examine AMEP412 activated biochemical responses, the ROS accumulation were detected using DAB and NBT, respectively. As shown in Fig. 5, with the increase of treatment time, brown DAB-stained and blue NBT-stained precipitates were increasing clearly observed. At 24 h post treatment, precipitates spread all over the tobacco leaves.

### Increase of defense enzymes in AMEP412 treated plants

**Fig. 6:**
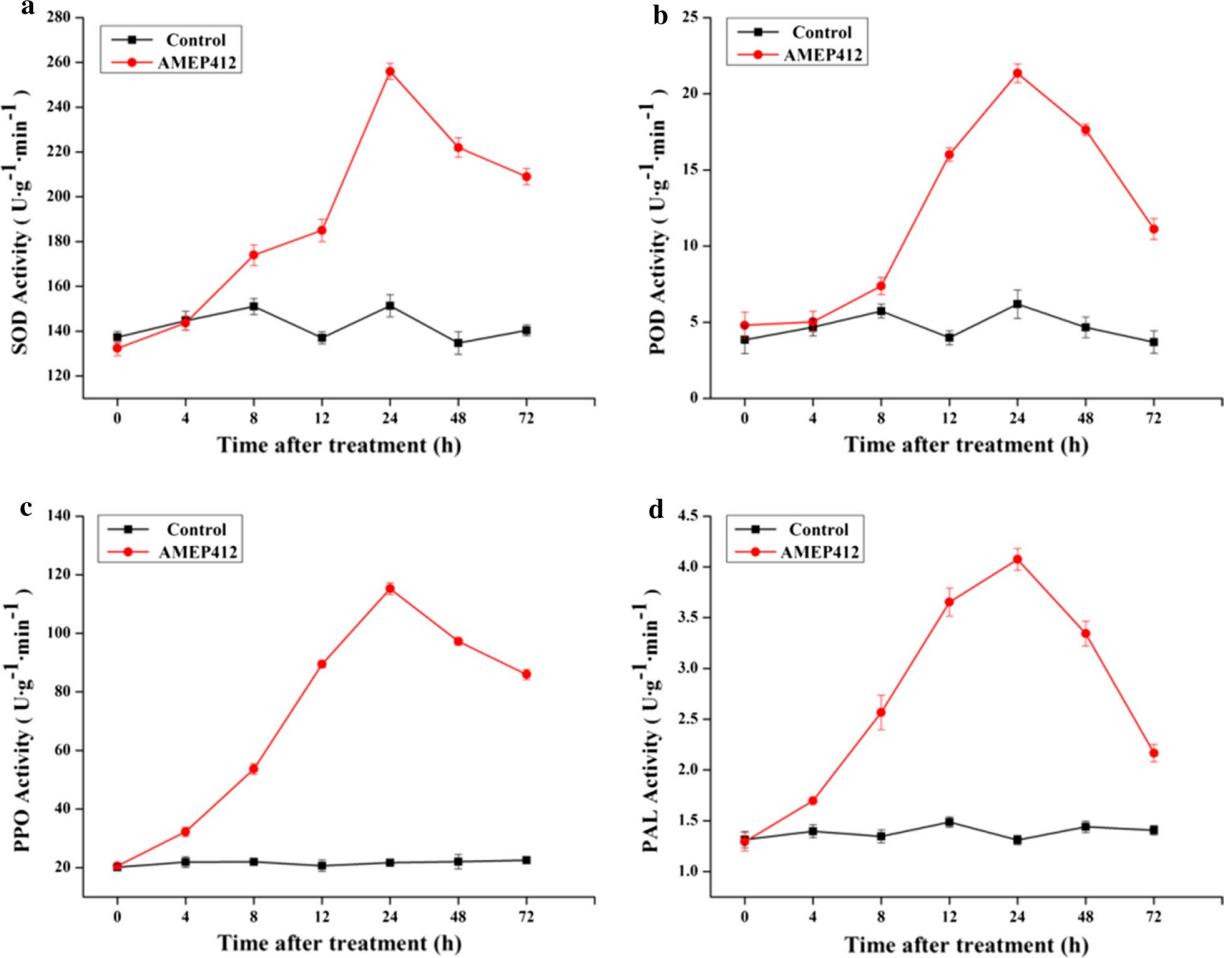
Kinetics of SOD, POD, PPO and PAL activity after AMEP412 treatment. The activities were measured 0–72 h after AMEP412 treatment. The values are the mean ± SD of quintuplicate samples

Defense related enzymes, including SOD, POD, PPO and PAL, were detected from 0 to 72 h after AMEP412 treatment. The activities of the above enzymes showed a similar trend, which appeared to be stimulated at 8 h, peaked at 24 h after AMEP412 treatment and then gradually declined in the AMEP412 treated plants (Fig. 6).

### AMEP412 induced plant SAR

**Fig. 7:**
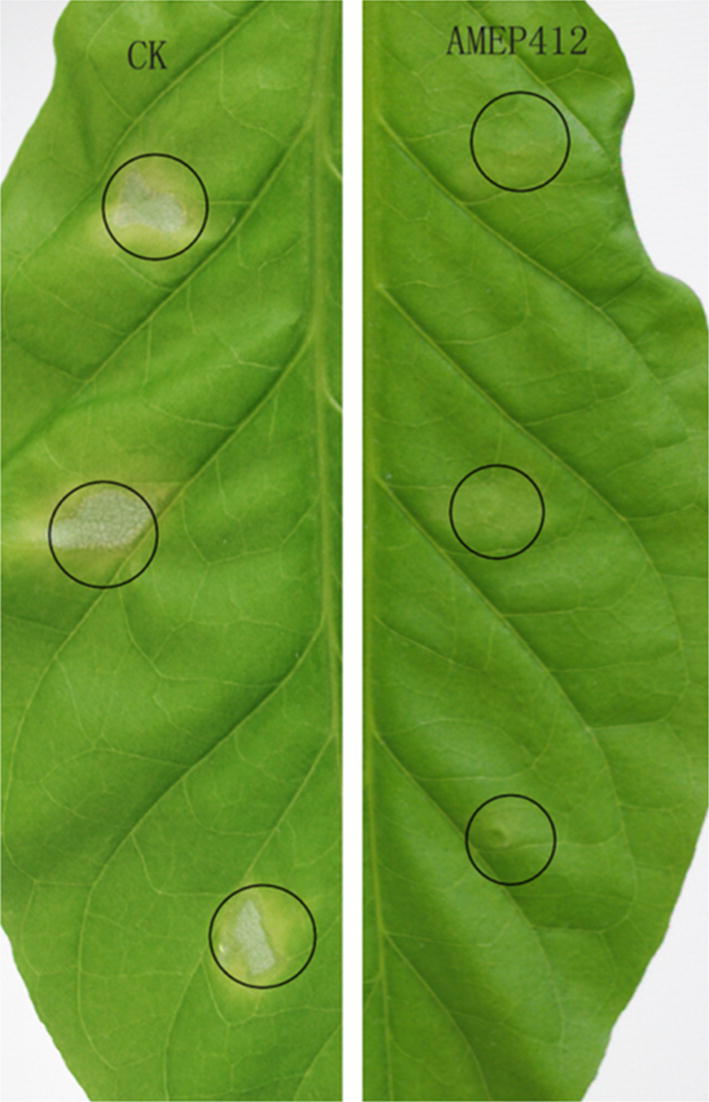
AMEP412 induced systemic resistance in tobacco against Pst DC3000

The ability of AMEP412 to induce SAR of *N. tabacum* against Pst DC3000 was tested. The AMEP412 treatment significantly inhibited the lesion caused by Pst DC3000 in area and severity (Fig. 7), which suggested that AMEP412 could induce plant SAR.

## Discussion

In this work, a novel protein elicitor AMEP412 was purified and characterized from *Bacillus subtilis* BU412. According to the BLAST result, AMEP412 was an uncharacterized protein without any function identified. It was reported widely distributed in genus *Bacillus*, including *Bacillus velezensis*, *Bacillus amyloliquefaciens*, *Bacillus vallismortis*, *Bacillus subtilis*, *Bacillus vietnamensis*, and *Bacillus aquimaris*. However, according to our research, the expression level of AMEP412 had an obvious dissimilarity between *Bacillus* strains (data not shown). It’s worth mentioning that *Bacillus subtilis* BU412 had a high expression level of AMEP412, which not only facilitated its purification and identification, but also provided material for further assays.

Different with most other elicitors, AMEP412 is secreted by *Bacillus subtilis*, which is regarded as an ideal biocontrol strain. Its advantage lies in that it can secret various antimicrobial peptides (AMPs) in the fermentation process, such as Surfactin (Peypoux et al. 1999), Fengycin (Hu et al. 2007), Bacilysin (Rajavel et al. 2009), and Iturin (Arrebola et al. 2010). All the encoding genes of these AMPs were successfully detected by PCR amplification using BU412 as template (data not shown). Unlike eliciting plants’ resistance against pathogens, these AMPs can directly inhibit plant pathogens. It will be a great enhancement for the disease control effect of AMEP412 applied products if these AMPs could be effectively saved during the fermentation and purification process.

AMEP412 contained 76 amino acid residues with a relative molecular mass of 8.36 kDa. However, according to the result of Superdex chromatography, the elution volume of AMEP412 (Fig. 1b, Peak F1) corresponded to a molecular weight of > 43 kDa, which was several times of its real molecular weight. This result indicated that AMEP412 probably formed polymers. The polymerization state could enhance its stability against thermal treatment, which explained why AMEP412 exhibited a good thermal stability.

HR is a form of cell death, which is regarded as part of plant innate immunity (Atkinson et al. 1990). Although some elicitors do not lead to HR symptoms (Mao et al. 2010; Zhang et al. 2010), HR is regarded as an important early event and widely used in elicitor screen and identification. In this study, high concentrations of AMEP412 could induce necrosis in tobacco leaves, and Trypan blue staining confirmed this result. Serial dilutions of AMEP412 were infiltrated into tobacco leaves for HR testing, and the minimum concentration of AMEP412 that inducing HR was 0.8 mg mL^−1^.

Low concentrations of AMEP412 could not cause visible HR symptom. However, they could still interact with tobacco leaves and trigger a serials of defence responses, like ROS accumulation and expression of defense enzymes, which leaded to the activation of the plant immune system (Dangl and Jones 2001; Chisholm et al. 2006). In our research, all these defence responses occurred at about 24 h after treatment. However, some elicitors required more than 2 days to show the responses (Zhang et al. 2010; Bu et al. 2013; Wang et al. 2016). The reason of the difference probably lied in the diversified mechanisms, which deserved clarification in the following research. Considering the degradation problem of elicitors in application, fast induction of defence responses should be a non-negligible advantage for commercial product.

In this research, the new elicitor was observed to be localized on the surface of tobacco cell, which indicated that it did not need to enter the cell to exert its function. So, it was predicted there might be some receptors interacted with the new elicitor and then transducted the signal into the cell through a certain pathway. It has been verified that salicylic acid (SA) and jasmonic acid (JA) signal pathways are responsible for the elicitor triggered plant defence responses. SA is a key regulator of SAR, whereas JA is indispensable for ISR (Spoel and Dong 2008). Moreover, interactions have been reported between these two pathways, which can be either antagonistic or synergistic. However, the antagonistic interactions seem to be dominant (Yang et al. 2015). For AMEP412, more researches are needed to reveal the receptor it interacts and the signal transduction pathway it utilizes.

In summary, our results showed that *Bacillus subtilis* BU412 produced a novel protein elicitor, AMEP412. It triggered a hypersensitive response in tobacco leaves and induced the production of signaling molecules and secondary metabolites related to plant resistance. Our results indicated that AMEP412 was a good plant defense activator and could be developed to a novel biopesticide in the future. In our next research, the fermentation conditions of BU412 will be optimized to increase the yield of AMEP412. Meanwhile, the exact mechanisms underlying AMEP412 induced priming of plant defence responses will also be studied.

## Additional file

**Additional file 1: Figure S1.** The mass spectrometry of the target protein. **Figure S2.** Amino acid sequence information of AMEP412 (WP_017418614.1). Amino acid sequence of AMEP412 in the one letter code. A predicted secondary structure is given in the lower line. H, Alpha helix; C, random coiled. Bold and italic letters, the peptide fragments detected by MS sequencing. Underlined, the predicted transmembrane domain.

## Data Availability

Please turn to authors for all requests.

## Abbreviations

HR: hypersensitive response
SAR: systemic acquired resistance
SOD: superoxide dismutase
POD: peroxidase
PPO: polyphenol oxidase
PAL: phenylalanine ammonia-lyase
ROS: reactive oxygen species
PR: pathogenesis-related
ISR: induced system resistance
SAR: systemic acquired resistance
PMF: peptide mass fingerprinting
DAB: 3,3-diaminobenzidine
NBT: nitro blue tetrazolium
