# Supplementary material for: Isolation and identification of a novel protein elicitor from a Bacillus subtilis strain BU412
Source: AMB Express. 2019 Jul 27;9:117. doi: 10.1186/s13568-019-0822-5 (PMC6661057; doi:10.1186/s13568-019-0822-5)

**Additional file 1:**

**Figure S1.** The mass spectrometry of the target protein

**Figure S2.** Amino acid sequence information of AMEP412 (WP_017418614.1). Amino acid sequence of AMEP412 in the one letter code. A predicted secondary structure is given in the lower line. H, Alpha helix; C, random coiled. Bold and italic letters, the peptide fragments detected by MS sequencing. Underlined, the predicted transmembrane domain

**Figure S1**


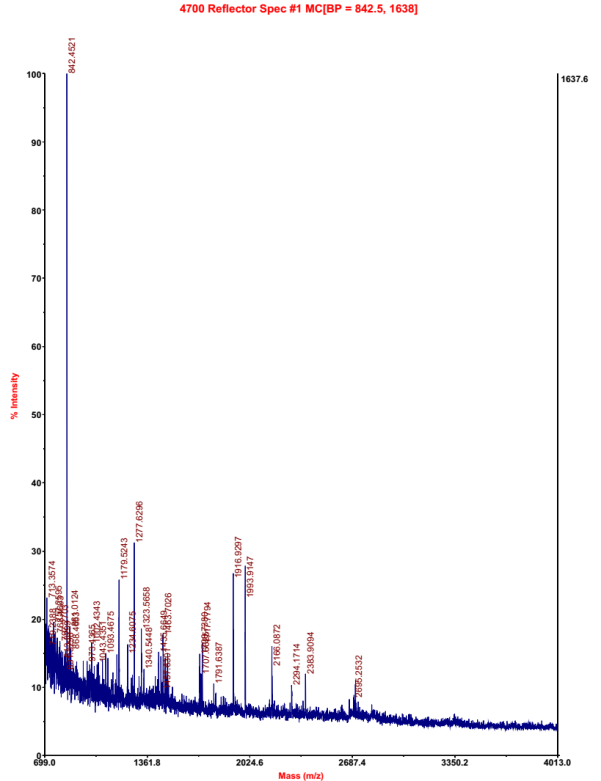


**Figure S2**


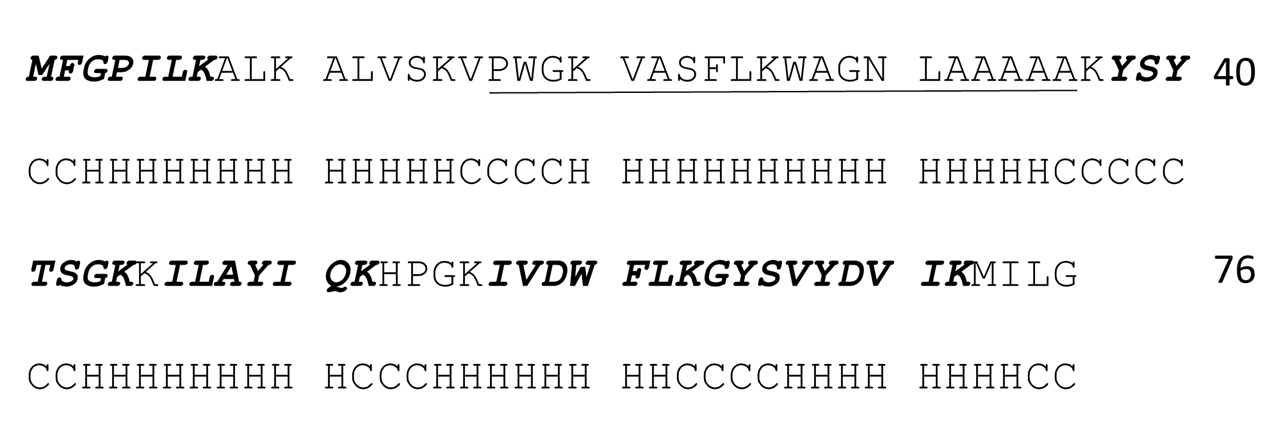

Supplement: Supplementary file 1 — Additional file 1: Figure S1. The mass spectrometry of the target protein. Figure S2. Amino acid sequence information of AMEP412 (WP_017418614.1). Amino acid sequence of AMEP412 in the one letter code. A predicted secondary structure is given in the lower line. H, Alpha helix; C, random coiled. Bold and italic letters, the peptide fragments detected by MS sequencing. Underlined, the predicted transmembrane domain. [file 13568_2019_822_MOESM1_ESM.docx]
